# Evaluation of Crossbreeding of Australian Superfine Merinos with Gansu Alpine Finewool Sheep to Improve Wool Characteristics

**DOI:** 10.1371/journal.pone.0166374

**Published:** 2016-11-10

**Authors:** Wenhui Li, Jian Guo, Fanwen Li, Chune Niu

**Affiliations:** 1 Gansu Provincial Sheep Breeding Technology Extension Station, Huang Cheng town, Sunan, Gansu, China; 2 Lanzhou Institute of Husbandry and Pharmaceutical Sciences, CAAS, Jiangouyan Street, Lanzhou, China; University of Florida, UNITED STATES

## Abstract

Crossbreeding of Australian Superfine Merinos (ASMs) with Gansu Alpine Finewool (GAF) sheep and an evaluation of the potential benefits of this genetic cross has not been previously conducted. 13 ASMs were crossbred with GAF sheep over a five year period with backcrossing designed to assess heterosis. Data from 11,178 lambs sired by 189 rams were used in the study. Genotype, birth year, birth type, dam age, sex and/or management group, and record age were fitted as fixed effects and within-genotype sire fitted as a random effect. Crossbreeds of 1/2 ASM expressed the most desirable effects for improving average fiber diameter (AFD), clean fleece weight (CFW), yield, coefficient of variation of AFD (CVAFD), yearling staple length (YSL) to AFD ratio (YSL/AFD), and CFW to metabolic yearling bodyweight (YWT^0.75^) ratio (CFW/YWT^0.75^) but showed the least post-weaning average daily gain (powADG) and YWT. Genotype of backcrossing with 1/4 ASM obtained moderate improvements in AFD, CFW, CVAFD, and YSL/AFD but the highest YSL, WWT, and prwADG. Except for yield (-1.42%) and CFW/YWT^0.75^ (-1%), heterosis estimates were generally low and positive, and ranged from 0.1% for CVAFD to 4% for powADG, which indicates the potential to improve relevant traits through exploiting heterosis to a varying extent. The ASMs sampled in this study were found to be superior to GAFs for AFD, CFW, yield, and CVAFD by 19.82%, 11.68%, 14.47%, and 6.99%, respectively, but inferior for YSL, PowADG, and YWT by 4.36%, 50.97%, and 16.93%, respectively. ASMs also appeared to be more efficient than GAFs in clean wool production (25.34%) and staple length growth (16.17%). The results of our study strongly suggest that an infusion of ASM genes via crossbreeding is an effective and appropriate approach to improve wool microns and wool production from GAF sheep, and we make recommendations to tackle the undesirable traits of YWT and YSL from ASM introduction.

## Introduction

Because of consumer demand for lighter weight fabrics and the severe challenge of competition from other synthetic fabrics, selective breeding of superfine wool sheep has been one of the most prevalent practices in the global fine wool sheep industry over the past 25 years. The significant achievements made in super and ultra-fine wool sheep breeding resulting from breeding programs like ' T13 ' [1] have put Australia in firm first place in the global fine wool sheep industry, and has resulted in the Merino enterprise producing wool in a more efficient manner. Although the total wool production figure in Australia decreased from 817,454 tons in 1991/92 to 365,561 tons in 2014/15, the production of superfine (≤18.5μm) increased 192%, and the proportion of superfine(≤18.5μm) wool production increased from 3.96% in 1991/92 to 25.87% in 2014/15 [2]. Adequate superfine/ultrafine wool sheep genetic resources have been made available due to these achievements in Australia. Meanwhile, there is practical and imperative need to develop superfine wool sheep breeds in China while maintaining adaptability to the environment. However, the challenge comes from the dearth of domestic superfine wool sheep genetic resources, and genetic progress in within-breed selection for reduced wool microns is known to have been slow.

Introducing Australian Merino genes into fine wool breeds is one of the dominant practices in fine wool sheep breeding in China. Notable numbers of Australian Merinos were imported in 1972, which was supposed to contribute to the development of Xingjiang Finewool sheep, and between 1984 and 1986, when 416 head (including 8 rams donated by the Australian government as part of the Australian Centre for International Agricultural Research project and used in the station of this study) were imported into China [3]. These genetic resources from Australia have made a significant contribution to the fine wool sheep gene pool in China, and previous researches on this issue have demonstrated that the introduction of the Australian Merino into different fine wool sheep breeds in China [4–5,7] and in America [6] has improved fleece weight, staple length, and clean fleece yield. During that period of time, imported Australian Merino rams were mainly of medium and strong wool type, and the goal of the crossbreeding them was to improve wool production while less attention was paid to improving wool fineness [4–5,7].

There is potential for Australian Superfine Merino (ASM) genetic resources to be used to improve the fine wool sheep breeds in other countries through crossbreeding. In a previous study, the Australian Merino involving rotational crosses were used to produce superfine wool [8]. Limited reports have been made available on the impact of introducing ASM genes into the local fine wool sheep breeds gene pool in China. For the Merino industry, the likely benefits of crossbreeding are in the expression of heterosis and a wider use of genetic resources [9].However, there needs to be caution when introducing new breeds since, while they may excel in a desirable trait, they are often inferior in other characteristics that contribute to overall merit of a sheep enterprise[10], and experiments designed to show which breeds and what methods of utilizing the better ones is one of the key requirements as to develop a more efficient animal industry while most effectively using world breed resources[11].

The Gansu Alpine Finewool (GAF) sheep is a dual purpose sheep breed that was developed in the high and cold Qilian mountainous pasture, Gansu province, China, where the altitude is between 2600m to 3500m above sea level. It was formally identified by the Gansu Provincial government in 1980. There are approximately 1.2 million head of this breed in China, mainly distributed in the Sunan and Tianzhu counties of Gansu province [12]. It is one of the most important fine wool sheep breeds in China and has underpinned the progressive development of that sector of the sheep industry in China for the past over three decades.

A program aimed at selecting superfine strain in GAF sheep commenced in the late 1990s. A hypothesis was made that introducing ASM genes into the GAF sheep gene pool would improve fleece quality, especially wool characteristics by affecting wool fiber diameter more effectively than within-breed selection. When carrying out a crossbreeding program, the expectation is, firstly, the infusion of new genes will change the genetic make-up of the existing population to the desirable phenotypic characteristics in the long term, and secondly, to utilize heterosis in the short term. It is necessary to differentiate the contribution of heterosis from that of the true breed genetic difference. Few articles in the literature have covered the knowledge in this field and previous studies have only evaluated the effects of crossbreeding of strong and medium Australian Merinos with Chinese local finewool sheep by way of comparing the performance of the crossbred progeny with the purebred local ones [4,5]. Nevertheless, estimates on heterosis in the crossbred progeny in these studies were not made for some reason.

13ASM rams were imported from Australia for an attempt to improve wool fineness and other characteristics by crossbreeding with GAF sheep, which commenced in 2005. The objective of this study is to evaluate the comprehensive effects of introducing ASM genes into the GAF sheep gene pool by crossbreeding. We test the hypothesis that the introduction of ASMs is an alternative to remedy the dearth of superfine Merino genetic resources in the Chinese domestic fine wool sheep population gene pool and give possible recommendations on the way that ASMs should be used in China.

## Materials and Methods

### Project Site

The project was carried out at Gansu Provincial Sheep Breeding Technology Extension Station located on the northern slopes of the Lenglong summit of the Eastern segment of the Qilian mountains, approximately 37°53’N and 101°45’E, Gansu province. It is a typically cold semi-arid alpine environment at an altitude of 2,600 to 3,500m. The station possesses the nucleus flock of 12,000 GAF sheep (relative to the whole 1.2 million population of the breed). Breeding, selection, and dissemination of high genetic merit animals from this nucleus is the core role the organization plays in the sheep industry in the Northern sector of China.

GAF sheep were developed by grading local Mongolian and Tibetan ewes with Xingjiang fine wool and Caucasian fine wool rams followed by self-breeding within F2 and/or F3 progeny flocks and then many years of selection. Since 1980, when the breed was formally recognized by the government of the province, continuous efforts have been made to improve growth, wool production, and quality while maintaining their adaptability to the high-and-cold environment in that area, mainly through within-breed selection and introduction of exotic breeds. On a number of occasions during 1980 to 2000, Merino-type rams were introduced into the nucleus population raised at the station: Australian medium and strong-wool Merino rams in 1986, New Zealand Merino rams in 1989, Xinjiang fine wool rams in 1992, and Chinese Merino rams from Inner Mongolia in 1995. These Merino gene infusions have played a singularly important role in the comprehensive genetic improvement of GAF sheep regarding wool production and quality performance.

### Crossbreeding Program

The crossbreeding program was conducted during 2005 to 2011 at Gansu Provincial Sheep Breeding Technology Extension Station. Thirteen ASM rams selected from two stud farms in Victoria were imported in 2005. Their average wool fiber diameter (AFD) was 14.88 μm (13.6 μm–16.2 μm) when they were one year old. The average clean fleece weight (CFW) recorded in 2006 at the first shearing in China was 5.42 kg (3.8 kg–7.33kg), the average clean fleece yield (yield) was 57.21% and the AFD was 18.36 μm; the above mentioned traits were superior to GAF rams of the same age, although their average body weight was 84.31 kg, which was 13.23 kg lower. Starting in 2005, crossbreeding was carried out between pure ASM rams and GAF ewes for four consecutive years and F1 crossbreeding progeny (AG) were obtained from four drops between 2006 and 2009. Ram lambs from F1 AG were selected from these drops and reared for breeding purposes from which 46 rams were backcrossed with purebred GAF ewes so that backcrossing progeny (AGG) were obtained during years 2008 to 2010. Meanwhile, GAF purebred breeding (GG) were routinely carried out during 2005 up to 2009 as control group. Information on a total of 11,178 progeny sired by 189 rams were involved in the study are shown in Table 1, which shows the investigated traits together with the associated data structure and the number of animals involved.

**Table 1:** Number of Animals and Data Structure for the AG, AGG, and GG Genotypes involved in the Investigation over Five Years.

Genotype	AG				GG					AGG			Total
Birth Year	2006	2007	2008	2009	2006	2007	2008	2009	2010	2008	2009	2010	
BWT	1371	1290	1438	594	1510	1649	1241	485	221	222	397	760	11178
WWT	1176	1137	1095	478	846	1360	971	361	150	155	305	491	8525
prwADG	1131	1137	1071	478	835	1360	949	361	150	155	305	491	8423
YWT	569	514	369	155	390	613	229	48	44	52	115	174	3272
powADG	542	495	345	146	327	582	213	45	39	48	107	161	3050
GFW	420	459	289	140	267	531	213	38	35	40	90	137	2659
CFW	111	143	69	40	55	103	44	12	13	14	18	31	653
Yield	153	181	89	45	95	150	59	16	18	16	24	40	886
AFD	341	272	185	64	249	379	74	24	38	22	25	127	1800
CVAFD	341	272	185	64	249	378	74	24	38	22	25	127	1799
YSL	569	514	369	155	390	613	229	48	44	52	115	174	3272
YSL/AFD	328	247	174	56	231	360	71	24	36	20	22	118	1687
CFW/YWT^0.75^	104	133	65	35	51	98	43	12	12	14	17	29	613

BWT, birth weight; WWT, weaning weight; prwADG, pre-weaning average daily gain; powADG, post-weaning average daily gain; YWT, yearling body weight; GFW, greasy fleece weight; CVAFD, coefficient of variation of average fiber diameter; YSL, yearling staple length; YSL/AFD, yearling staple length to average fiber diameter ratio; CFW/YWT^0.75^, clean fleece weight to metabolic yearling body weight ratio; AG, ASMx GAF; AGG, AG x GAF; GG, GAF purebred.

### Sheep Management and Traits Measurement

The experimental ewes were from six ewe breeding flocks (BFs), the size of which were approximately 750 except for one which had only 500. The mobs of ewes were grazed on similar pastures and the same supplementary feeding and management system was applied. Trans-cervical fresh semen artificial insemination (AI) was applied once a year between November 20th and December 10th, after which the back-up rams routinely joined with the ewes for another 20 days. At commencement of the AI program, vasectomized teaser rams were used twice daily (morning and afternoon) for estrus detection. The estrus ewes were drafted out and allocated to mating groups of 15–20 ewes. AI was conducted each morning and individual ewes were inseminated once per day on two consecutive days.

The corresponding lambing period took place from mid-April and continued until the end of May. Lambs were weighed at birth, ear-tagged and the pedigree recorded within 24 h of birth. Birth year (BY), sex, birth type (BT, single or twin) and dam age (DA, adult or maiden) were also recorded. Weaning and weaning assessment was conducted at an average age of 115 days, when weaning weight (WWT) was recorded. Pre-weaning average daily gain (prwADG) was calculated as the product of the difference between WWT and BWT divided by the number of weaning days. After weaning, animals were managed under post-weaning sex and/or management groups (SG: male1, male2, and female).

From approximately 4,500 male weaners, 70–90 ram lambs were selected each year as breeding rams and formed a special rearing flock (male1). These rams were supplementary fed from October to the following May inclusively (approximately 210kg of concentrate and 120kg of oat hay per head). A further 300–400 ram lambs were selected to form a common rearing flock (male2) and were also supplementary fed but at a lower rate than the special rearing group (approximately 46kg of concentrate and 50kg of oat hay per head from January to May). Approximately 2,100 out of 4,500 ewe lambs were selected each year as replacement ewes and were managed in two or three groups (female) with the same supplementary feeding regime as the common rearing ram lambs. Selection of both ewe and ram weaners were mainly based on weaning weight and visual scores of wool characteristics; surplus lambs were sold.

Yearling bodyweight (YWT) and yearling mid-side wool staple length (YSL, measured with a steel ruler to the nearest 0.5cm) were obtained on retained animals in the middle of June in the next year at yearling assessment when the animals were 13–14 months old. Post-weaning average daily gain (powADG) was calculated as the product of the difference between YWT and WWT divided by rearing days between weaning and yearling assessment. Mid-side wool samples were also taken randomly from all the yearling animals classed as ‘top’ grade and ‘first’ grade with clear identification in an attempt to keep similar numbers of animals in different subgroups at this time to test the yield, AFD, and coefficient of variation of mean fiber diameter (CVAFD). Approximately 20 days after assessment, all the sheep were shorn and individual greasy fleece weight (GFW) recorded. CFW was calculated as the product of GFW multiplied by the yield. Two secondary traits were used in the investigation, they are respectively the ratio of clean fleece weight to metabolic yearling body weight (CFW/YWT^0.75^) and staple length to average fiber diameter (YSL/AFD) ratio. Since CFW and YWT were recorded at different ages, they were initially adjusted to the same record age 435 days before calculating the final CFW/YWT^0.75^ ratio.

All experimental and surgical procedures were approved by the Institutional Animal Care and Use committee, Lanzhou Institute of Husbandry and Pharmaceutical Sciences, People’s Republic of China. All efforts were made in animal handling to minimize suffering during AI and other activities.

### Statistical Analysis

The data were analyzed using general linear model realized with ASReml-3 [13]. The statistical model included genotype (cross AG, backcross AGG, and purebred GG), SG, BY, BT, DA, and BF as fixed effects. BF was included in pre-weaning but excluded from post-weaning traits. Age at trait recording was fitted as a covariate for WWT, YWT, YSL, GFW, CFW, and YSL/AFD. The within-genotype sire effect was treated as a random effect. Sire effects were preliminarily included in the model and then excluded when the effects were found to be non-significant. The significance of the sire effect was analyzed by log-likelihood ratio testing with χ2– twice the log-likelihood difference between the model without and the model with the sire effect. The significance of the random effect was tested at P<0.05 by comparing the differences in log-likelihood with values for a χ2 distribution with three degrees of freedom. Two-way interactions among BT, SG, BY, DA, and BF were preliminarily fitted in the linear model and then those found to be non-significant were excluded. Under this general linear model, each individual effect was expected to function independently of the others.

### Estimates of Heterosis and True Breeding Value Comparison between two Breeds

Backcrossing was designed to estimate the heterosis of the two breeds. 46 F1 AG sires were used to mate with purebred GAF sheep. In the backcross, only half of the gene pairs involved a difference in breed of origin. Thus, heterosis expression is 50%. Accordingly, Heterosis (%) = 50(2 x AGG/(AG+GG)-1).

Based on the theory that if there is no heterosis (H) between the two breeds, the difference in the predicted breeding value between the two breeds is simply double the difference between the crossbred and purebred GAF sheep; in the current case, Breeding value difference = 2(AG/(H+1)-GG)

## Results

Predicted means and standard errors of five growth traits: BWT, WWT, prwADG, powADG, and YWT are presented in Table 2, while that of the traits representing wool characteristics: GFW, AFD, CFW, yield, YSL, CVAFD, and two derived relative traits YSL/AFD and CFW/YWT^0.75^ are presented in Table 3. Overall means were adjusted to be single-born from adult ewes for the traits which were significantly influenced by BT and DA, and powADG and wool traits were additionally adjusted to be reared in the female post-weaning management group.

**Table 2:** Predicted Means (± Standard Error) for Fixed Effects plus the Significance of any Two-way Interactions.

Source of variation	BWT(kg)	WWT (kg)	prwADG (g/d)	powADG (g/d)	YWT
**Overall Mean**	3.81 (0.01)	24.67 (0.09)	180.05 (0.74)	37.90 (0.46)	36.57 (0.14)
**Genotype (GT)**	n.s.	***	***	***	***
AG	3.78 (0.03)	24.60 (0.12)ab	178.77 (0.97)a	32.11 (0.72)a	34.86 (0.23)a
GG	3.82 (0.02)	24.46 (0.15)a	177.71 (1.21)a	41.44 (0.55)b	37.44 (0.20)b
AGG	3.83 (0.02)	24.990 (0.165)b	184.08 (1.48)b	39.72 (1.15)b	37.38 (0.29)b
**Birth Year (BY)**	***	***	***	***	***
2006	3.66 (0.02)c	23.60 (0.14)d	165.33 (0.98)d	34.14 (0.55)c	35.13 (0.18)b
2007	3.89 (0.02)a	25.33 (0.11)a	184.10 (0.89)ab	27.69 (0.52)d	33.92 (0.17)a
2008	3.88 (0.02)a	24.97 (0.15)b	184.74 (1.22)a	42.86 (0.82)b	38.47 (0.24)d
2009	3.90 (0.02)a	24.99 (0.16)ab	181.38 (1.39)bc	34.64 (0.97)c	36.00 (0.29)c
2010	3.70 (0.03)c	24.29 (0.22)c	180.26 (1.80)c	49.30 (1.17)a	38.64 (0.34)d
Sex (Sex Group, SG)	***	***	***	***	***
male1	3.88 (0.01)	25.26 (0.11)	184.30 (0.88)	118.83 (0.91)a	66.41 (0.28)a
male2				51.60 (0.85)b	44.41 (0.25)b
female	3.74 (0.01)	24.08 (0.10)	175.75 (0.85)	37.90 (0.46)c	36.57 (0.14)c
BirthType (BT)	***	***	***	**	n.s.
single	3.82 (0.01)	24.67 (0.09)	180.05 (0.74)	37.90 (0.46)	
twins	2.92 (0.03)	21.94 (0.27)	163.17 (2.23)	41.36 (0.16)	
Dam Age (DA)	***	***	***	***	n.s.
maiden	3.36 (0.02)	21.71 (0.17)	157.68 (1.29)	42.38 (1.00)	
adult	3.82 (0.01)	24.67 (0.09)	180.05 (0.74)	37.90 (0.46)	
Birth Flock (BF)	***	***	***	—	—
**Record Age**	NA	116.7***	NA	NA	n.s.
**Sire**	***	***	***	***	***
BT×DA	**	n.s.	n.s.	n.s.	n.s.
BT×BF	**	***	***	—	—
BT×GT	n.s.	*	*	n.s.	n.s.
BT×BY	n.s.	***	***	n.s.	n.s.
DA×SG	n.s.	*	n.s.	n.s.	n.s.
DA×BY	n.s.	**	n.s.	n.s.	n.s.
SG×BY	**	*	n.s.	***	***
SG×BF	n.s.	*	*	—	—
BF×GT	***	***	***	—	—
BF×BY	***	n.s.	n.s.	—	—
GT×BY	n.s.	***	***	***	**

BWT, birth weight; WWT, weaning weight; prwADG, pre-weaning average daily gain; powADG, post-weaning average daily gain; YWT, yearling body weight;AG, ASMx GAF; AGG, AG x GAF; GG, GAF purebred. Predicted overall means and means of fixed effects were adjusted to single-born from adult ewe for pre-weaning growth traits, while they were additionally adjusted to manage under female sex and/or management group for post-weaning growth traits. In addition to the above adjustments, weaning weight was adjusted to the average of 116.7 days of age.

***P<0.001;

**P<0.01;

*P<0.05;

n.s. not significant. Means with different levels within effects followed by the same letters are not significantly different (at P = 0.05).

**Table 3:** Predicted Means (± Standard Error) for Wool Traits plus Significance of Two-way Interactions of Fixed Effects.

Source of variation	GFW (kg)	AFD (μm)	CFW (kg)	Yield (%)	YSL (cm)	CVAFD (%)	YSL/AFD (cm/ μm)	CFW/YWT^0.75^ (g/kg)
**Overall Mean**	3.79 (0.03)	16.95 (0.12)	2.03 (0.03)	54.94 (0.33)	10.16 (0.04)	21.13 (0.13)	0.618 (0.003)	130.71(1.29)
**Genotype (GT)**	n.s.	***	*	***	*	*	***	***
AG	3.72 (0.04)	16.00 (0.13)a	2.09 (0.03)a	57.02 (0.44)a	10.04 (0.05)a	20.94 (0.19)a	0.644 (0.003) a	139.17 (1.50) a
GG	3.73 (0.04)	17.72 (0.14)c	1.95 (0.05)b	53.93 (0.40)b	10.13 (0.06)a	21.68 (0.22)c	0.592 (0.004) c	124.77 (1.86) b
AGG	3.78 (0.07)	16.93 (0.19)b	2.09 (0.08)ab	53.91 (1.05)b	10.37 (0.07)b	21.35 (0.33)ab	0.626 (0.007) b	129.32 (3.07) b
**Birth Year (BY)**	***	***	***	***	***	***	***	***
2006	3.33 (0.04)d	17.69 (0.13)ab	1.86 (0.04)b	56.90 (0.43)a	9.89 (0.05)a	23.33 (0.17)a	0.580 (0.004) de	125.68 (1.92) c
2007	3.46 (0.03)c	16.32 (0.13)d	1.92 (0.04)b	55.33 (0.36)b	9.93 (0.04)a	22.92 (0.17)b	0.620 (0.003) bc	130.88 (1.70) b
2008	4.26 (0.04)a	16.11 (0.15)d	2.18 (0.05)a	51.72 (0.55)c	10.25 (0.06)b	19.14 (0.24)d	0.657 (0.006) a	134.30 (2.26) ab
2009	3.90 (0.06)b	17.11 (0.18)c	2.14 (0.07)a	58.05 (0.74)a	10.38 (0.08)b	20.88 (0.34)c	0.624 (0.009) b	138.63 (2.97) a
2010	3.74 (0.07)b	17.87 (0.17)a	1.92 (0.09)b	52.77 (1.00)c	10.20 (0.09)b	20.51 (0.29)c	0.586 (0.007) d	118.29 (3.71) c
**Sex (Sex Group, SG)**	***	***	***	***	***	***	n.s.	***
male1	6.76 (0.05)a	17.68 (0.33)a	3.20 (0.05)a	50.26 (0.61)a	10.63 (0.07)a	18.81 (0.34)a	0.584 (0.007)	125.50 (2.16) b
male2	3.98 (0.08)b	16.07 (0.37)c	2.03 (0.09)b	55.11 (0.52)b	10.15 (0.07)b	22.07 (0.67)b	0.583 (0.016)	112.84 (3.66) c
female	3.79 (0.03)c	16.95 (0.12)b	2.03 (0.03)b	54.94 (0.33)b	10.16 (0.04)b	21.13 (0.13)b	0.618 (0.003)	130.71 (1.29) a
**Birth Type (BT)**	**	n.s.	n.s.	n.s.	n.s.	n.s.	n.s.	n.s.
single	3.79 (0.03)	16.65 (0.06)	—	54.94 (0.33)				
twins	3.46 (0.07)	17.25 (0.21)	—	57.19 (1.20)				
**Damage (DA)**	n.s.	n.s.	n.s.	*	n.s.	n.s.	n.s.	n.s.
maiden	—	—	—	56.98 (0.81)				
adult	—	—	—	54.94 (0.33)				
**Record Age**	434.8***	NA	435.3**	NA	415.4***	—	415.0***	—
**Sire**	***	***	**	**	***	***	n.s.	n.s.
BT×GT	n.s.	n.s.	n.s.	**	n.s.	n.s.	n.s.	n.s.
BT×SG	n.s.	**	n.s.	n.s.	n.s.	n.s.	n.s.	n.s.
SG×BY	***	n.s.	**	***	***	**	n.s.	n.s.
SG×GT	*	n.s.	n.s.	*	n.s.	n.s.	***	n.s.
GT×BY	n.s.	n.s.	n.s.	n.s.	*	**	n.s.	n.s.

GFW, greasy fleece weight; CVAFD, coefficient of variation of average fiber diameter; YSL, yearling staple length; YSL/AFD, yearling staple length to average fiber diameter ratio; CFW/YWT^0.75^, clean fleece weight to metabolic yearling body weight ratio; AG, ASMx GAF; AGG, AG x GAF; GG, GAF purebred.Predicted overall means and means of fixed effects were adjusted to manage under female sex and/or management group for all wool traits, adjusted to be single-born for GFW, and were additionally adjusted to be born from adult ewe for yield. In addition to the above adjustments, GFW, CFW, YSL, and YSL/AFD were respectively adjusted to averages of 434.8, 435.3, 415.4, and 415 days of age.

***P<0.001;

**P<0.01;

*P<0.05;

n.s. not significant. Means with different levels within effects followed by the same letters are not significantly different (at P = 0.05).

### Non-genetic Fixed Effect

All growth traits were significantly influenced by BY, SG, BT, and DA (P<0.01) except that YWT was not significantly influenced by either BT or DA. BF had a significant influence on BWT, WWT, and PrwADG. BY and SG significantly influenced all five wool traits and two derived relative traits with the exception that SG had no significant effect on YSL/AFD in this investigation. BT had no significant effect on all wool traits except GFW. DA only had a significant effect on yield but not on other wool traits. As shown in Tables 2 and 3, age at the time of recording had a significant influence on WWT, YWT, GFW,CFW, and YSL/AFD. Results of all two-way interactions with their significant test are respectively presented in Table 2 for growth traits and in Table 3 for wool traits and two derived relative traits.

### Effect of Genotypes

Comparisons in Tables 2 and 3 of the traits in the progeny from three genotypes: ASM x GAF (AG), GAF purebred (GG), and AG x GAF (AGG) show the effects of crossbreeding. Except for BWT and GFW, all growth, wool, and relative traits were influenced significantly by genotype. Within-genotype sire, as a random effect, significantly affected all growth traits and wool traits involved in this study but not the two relative traits. We observed the highest WWT (24.99±0.165 kg) and PreADG (184.08±1.48 g/d) for the AGG group, which were significantly higher than the GG group for WWT and the AG and GG groups for PreADG. No significant differences between the AG and GG groups were observed for the WWT and PreADG traits. The lowest post-weaning traits: PowADG (32.11±0.72 g/d) and YWT (34.86±0.23 kg) were observed for the AG group, both of which were significantly lower than the GG and AGG groups. No significant differences were found between GG and AGG groups for PowADG and YWT.

The lowest AFD was recorded for the crossbreed AG group (16.00±0.13μm) and purebred GG was the highest, while the backcross AGG group was in the middle. The three genotypes were significantly different from one another for AFD. For CVAFD, the AG group was observed to be the lowest and so the most desirable, while the GG group showed the highest and the AGG group was in the middle. The two crossbred genotypes showed significantly improved CVAFD over the purebred GG group, and no significant differences were found between themselves. Although no significant differences were found between the three genotypes for GFW, the AG group produced 0.14kg (P<0.05) more clean fleece weight than the GG group. No significant differences were found either between AG and AGG, or between GG and AGG, for CFW. Highest yield (57.02±0.44%) was observed for AG compared to GG and AGG while the latter two groups showed a similar yield. AGG realized the highest YSL(10.37±0.07cm, P<0.05) compared to AG and GG with no significant difference found between the latter two groups.

AG was observed to have the highest YSL/AFD, with AGG in the middle and GG the least, and were all significantly different from one another between the genotypes. The highest CFW/YWT^0.75^ was recorded for AG, GG was the lowest, and AGG in the middle. AG showed significant higher CFW/YWT^0.75^ than the other two groups, between which no significant difference was found for the trait.

The within-genotype sire group had a significant influence on all growth traits and wool traits involved in the study except for the two derived relative traits (shown in Tables 2 and 3). Genotype x BF significantly influenced BWT, WWT, and prwADG at the 0.1% significance level, and genotype x BY interaction had a significant effect on WWT (P<0.001), prwADG (P<0.001), powADG (P<0.001), YWT (P<0.01), YSL (P<0.05), and CVAFD (P<0.01). Genotype x BT had a significant effect on WWT (P<0.05), PreADG (P<0.05), and yield (P<0.01). The SG x genotype interaction was observed to have a significant influence on GFW (P<0.05), yield (P<0.05), and YSL/AFD (P<0.001).

### Estimates of Heterosis and Comparison between Breeds

From Table 4, we can see that desirable positive estimates of heterosis were observed for CFW (1.72%) and GFW (0.71%), while undesirable negative heterosis for yield (-1.42%) and positive but low heterosis for AFD (0.21%) and CVAFD (0.1%) were recorded in this study. Desirable positive estimates of heterosis for growth traits were observed, the magnitude of which ranged from 0.42% for BWT (the lowest) to 4% for powADG (the highest). Heterosis estimates were desirable and positive for trait YSL/AFD but undesirable and negative for trait CFW/YWT^0.75^. Means of the purebred ASM population were estimated by excluding the extent of heterosis from F1 crossbred performances. Hence, this allowed us to compare the true genetic differences between the two breeds (shown in Table 4). ASMs sampled in this study were superior to GAF sheep for AFD (-3.5μm:-19.82%), yield (7.81%: 14.47%), CFW (0.23kg: 11.68%), CVAFD (-1.51%: -6.99%), YSL/AFD (0.096 cm/μm: 16.17%), and CFW/YWT^0.75^(31.62 g/kg: 25.34%). However, ASMs appeared to have less YSL (-0.44cm: -4.36%) and GFW (-0.07kg: -1.77%) than GAF sheep. They also showed inferior BWT (-0.11kg:-2.93%), WWT (-0.18kg: -0.72%), prwADG (-3.65g/d:-2.06%), powADG (-21.12g/d:-50.97%), and YWT (-6.34kg:-16.93%) to GAF sheep.

**Table 4:** Estimation of Heterosis and Comparison of True Breed Difference.

Traits	Average of AG +GG	Means of AGG	Heterosis (%)	Estimated means of Purebred ASM	Difference (ASM-GAF)	
					Absolute	Relative (%)
**Wool Traits**						
AFD (μm)	16.86	16.93	0.21%	14.211	-3.51	-19.82%
GFW (kg)	3.72	3.78	0.71%	3.661	-0.07	-1.77%
Yield (%)	55.48	53.91	-1.42%	61.738	7.81	14.47%
CFW (kg)	2.02	2.09	1.72%	2.172	0.23	11.68%
YSL (cm)	10.09	10.37	1.39%	9.685	-0.44	-4.36%
CVAFD (%)	21.31	21.35	0.10%	20.163	-1.51	-6.99%
**Growth Traits**						
BWT(kg)	3.80	3.83	0.42%	3.704	-0.11	-2.93%
WWT (kg)	24.53	24.99	0.94%	24.283	-0.18	-0.72%
prwADG (g/d)	178.24	184.08	1.64%	174.055	-3.65	-2.06%
powADG (g/d)	36.77	39.72	4.00%	20.315	-21.12	-50.97%
YWT (kg)	36.15	37.38	1.70%	31.106	-6.34	-16.93%
**Relative traits**						
YSL/AFD (cm/μm)	0.618	0.626	0.647%	0.688	0.096	16.17%
CFW/YWT^0.75^ (g/kg)	131.97	129.32	-1.004%	156.39	31.62	25.34%

Heterosis (%) = 50(2 x AGG/(AG+GG)-1), estimated means of purebred ASM = 2 x AG/(H+1)–GG, and, therefore, the true breed difference(ASM-GAF) = 2(AG/(H+1)-GG).BWT, birth weight; WWT, weaning weight; prwADG, pre-weaning average daily gain; powADG, post-weaning average daily gain; YWT, yearling body weight; GFW, greasy fleece weight; CVAFD, coefficient of variation of average fiber diameter; YSL, yearling staple length; YSL/AFD, yearling staple length to average fiber diameter ratio; CFW/YWT^0.75^, clean fleece weight to metabolic yearling body weight ratio; AG, ASMx GAF; AGG, AG x GAF; GG, GAF purebred.

## Discussion

### The Impact of ASM Genotype Level

The paucity of superfine Merino genetic resources is one of the key obstacles that hinders the transformation of the fine wool industry in China into a more sustainable and efficient state. Our study gave testimony to the hypothesis that introducing ASMs and crossing them with Chinese Merino-type fine wool sheep is an option to resolve the issue, and we have provided useful information on the way to introduce ASM genes into the GAF sheep gene pool. The best performance displayed by the crossbred AG genotype in the investigation of wool traits (AFD, CVAFD, Yield, CFW, YSL/AFD, and CFW/YWT^0.75^) showed that this crossbreeding was mainly a success. However, undesirable results were observed for the PowADG and YWT traits. Previous research has reported that the crossbreeding of Australian Merinos with GAF sheep resulted in desirable improvement of CFW and yield [7], and similar improvements were also observed for CFW and yield with Xinjiang Finewool sheep [4] and Inner Mongolian Finewool sheep [5]. These researches also reported improvement for YSL with no significant decrease of YWT. An apparent reason for this disagreement may be that the Australian Merino rams used in the current study were superfine Merino stock while those used in the previous studies were of strong and medium wool-type [4,5,7]. On the other hand, the backcross genotype (AGG) with 1/4 ASM was generally observed to have the best performance in YSL among the three genotypes, and to be superior in WWT, PrwADG, AFD, and CVAFD over purebred GAF sheep, and similar PowADG, YWT, CFW, yield, and CFW/YWT^0.75^ with GG genotype. We found an improving trend for AFD, CVAFD, YSL/AFD, and CFW/YWT^0.75^ with increasing Australian Merino inheritance, which was also found in terms of yield and AFD when crossing Australian Merino with Rambouilleit sheep in a previous research [14].

The improvement of AFD brought about by the infusion of ASM genes is generally consistent with what was expected in the planning of the crossbreeding program. If we assume that there was no heterosis present in the crossbred progeny, the decrease of AFD will generally bring about a negative change in CFW and body weight of the animal because it is widely accepted that AFD is positively genetically correlated with CFW and body weight. Weighted means of genetic correlation between AFD and post-weaning body weight and adult body weight are 0.20 and 0.15, respectively, and that between AFD and CFW is 0.28 [15].

The positive genetic correlation between AFD and YWT could be a reasonable explanation for the inferior expression of growth traits in the crossbred genotype. As the inheritance of ASM decreases in the backcross AGG genotype, growth performance was considerably improved, which implies that the inferior performance of the growth traits in the crossbred genotype may, firstly, partially come from the additive genetic contribution of ASM genes, and, secondly, partially come from the adaptive inferiority of ASMs to the local environment or from the breed x environment interaction. However, the positive genetic correlation between AFD and CFW cannot explain the results found in this investigation that the crossbreeding not only improved AFD but also simultaneously improved CFW, especially in cases where YWT decreased.

Wool production is generally believed to depend on wool fiber density; skin area, which is closely related to body weight; and wool staple length. As there was a decrease in body weight and not much difference in staple length between the crossbred and purebred GAF sheep, the superior CFW realized in the AG genotype may be a consequence of the possibly increased fiber density, direct information on which we did not record in the current study.

In a previous study, researchers argued that the decrease in body weight (and hence the surface area) in animals with lower fiber diameter is too small to account for the increases observed in follicle density [16]. They concluded that selection for reduced fiber diameter may decrease live weight, and finer diameter may affect nutrient metabolism through two adaptations that tend to maintain fleece weight: an increase in follicle density and/or an increase in relative fiber length. In this investigation, with an increase of inheritance from the ASMs, the AFD decreased but relative fiber length increased, which is in conformity with previous findings [16].

The superior CFW/YWT^0.75^ ratio observed in genotypes with ASM genes demonstrated that the Australian Merino is a more efficient wool producer than GAF, as has been similarly reported when Australian Merinos were crossed with the Polwarth breed [17]. However, what we have to keep in mind is that the economic advantage of a breed depends largely on the price advantage of finer wool compared to the advantage of the price of larger hoggets [17]. In these dry, cold, and harsh highland pastures, animals with relatively large body weight are more welcomed than small sized animals.

Except for two relative traits, significant within-genotype sire effects for most of the growth and wool traits implies that there is potential for improving growth and wool traits through exploiting variation amongst sire progeny groups while carrying out crossbreeding programs. These variations may come from the genetic differences between the individual sires, so more emphasis should be put on selecting breeding rams in a specific Merino production system.

Inclusion of the various fixed effects with their interactions in the model was mainly to allow for the assumption on the effect of genotype function independent of others. The significant year effect found in the study for all traits was apparently a reflection of varying climate and pasture conditions during the years of the study. The effect of SG mainly resulted from different retention rates at weaning and different rearing conditions during the post-weaning period. The results showed that the superior rearing conditions in breeding rams (male1) resulted in a notable decrease of variation of average wool fiber diameter since it may reduce the variation of fiber diameter along wool staple, especially when the rams received more supplementary feeding during dry and cold winter periods. On the other hand, significantly lower yields observed in the breeding ram group mainly resulted from the fact that these animals spent more time staying in or around the shed to get more supplementary feed, and so there was more chance of their wool becoming contaminated than the two other groups. The effect of DA and BT indicates that lambs born as twins and born to maiden dams were restricted in pre-weaning growth, but they can display compensatory growth post-weaning. Similar fixed effects were also reported in research based on the same population of GAF sheep [12].

### Impact of Heterosis and True Breed Difference

There is limited information on heterosis estimates for the crossing of Australian Merinos with Chinese Merino-type finewool sheep in the literature. The major challenge for estimating heterosis has been mainly because of the fact that the performance of the exotic Australian Merino purebred could not be obtained in the imported environment, and the backcrossing has not specifically designed to deliberately obtain heterosis between the two breeds. Attempts were made to evaluatethe true breed difference of Australian Merino and Xinjiang fine wool sheep assuming that the heterosis was similar with the estimates between strains or bloodline of Australian merino, which was obviously inadequate to give a precise evaluation result [4].The heterosis estimates in our study generally agreed with the conclusion that heterosis for growth traits and wool production is usually in the range of 1% to 10% in crossbreeding Australian Merino strains or bloodlines [18]. The current estimates of heterosis are within the range of estimates between different strains of Australian Merino for traits YWT, yield, and CFW except that the estimates for AFD was much lower than in other studies (0.8% [19], 1.2% [20]). The negative direction of heterosis for yield (-1.42%) was also consistent with the results of these studies (-0.3% [19], -4.8% [20]). Our estimates of heterosis for GFW (0.71%) and WWT (0.94%) were much lower than that of the results obtained from crossbreeding Australian Merino with Polwarth sheep [17], which were respectively 2–3% for GFW and 10% for WWT. Interestingly, the direction of heterosis estimates for the CFW/YWT^0.75^ ratio in our study (-1%) is similar with that (-2%) previously reported [17].

Except for the post-weaning growth rate (4%), the heterosis estimates of all other traits are relatively low (-2% ~2%). One of the reasons for this is that, before this crossbreeding program commenced in 2005, Australian Merino genes had already been infused into the gene pool of this nucleus population of GAF sheep on a number of occasions. For example, purebred strong and medium wool Australian Merino rams were introduced in 1986, and some Xinjiang Finewool rams with Australian Merino blood were introduced in 1992, and so on. These Australian Merino gene infusion into the GAF sheep gene pool may have brought the genetic distance between the two breeds closer. This argument is supported by the research results of crossbreeding Australian Merino with a number of finewool sheep breeds in Inner Mongolia where heterosis was found to be lower in genotypes to which Australian Merino genes had been previously introduced than in those to which the genes were being introduced for the first time [5].

As is well-known, crossbreeding strategy has been widely adopted to exploit heterosis in the short term for the improvement of the targeted sheep population. Our study implies that there is some potential in exploiting heterosis through crossbreeding ASMs with GAF sheep. More importantly, heterosis estimation provide information on which the true breed difference resulted from additive genetic effect can be calculated. A better knowledge of both superiority and inferiority of the exotic breed to the local breed will help design a more effective and reliable crossbreeding program. As heterosis cannot be passed on to the next generation by interbreeding, what a sheep breeder expects from the introduction of exotic breed genes in the long run is to benefit from the additive genetic effect of the exotic breed. The true breed differences will surely provide valuable information for designing crossbreeding programs between GAF and ASM sheep.

#### Wool fiber diameter and its coefficient of variation

The estimated breed superiority of ASM over GAF sheep sampled for AFD (-19.82%) and CVAFD (-6.99%) in this study implies that the improvements of the two traits in the genotypes with ASM inheritance were mainly resulted from the additive genetic effects of the infused ASM gene. The heterosis of the two traits were undesirable but very low, hence can be ignored. Previous research has demonstrated the coefficient of variation of fiber diameter is negatively genetically related to staple strength (-0.46 to -0.86 [21]; -0.52 [15]).Researchers have previously reported that the correlation between sire estimated breeding values for coefficient of variation of fiber diameter (CVAFD) measured at a hogget shearing and staple strength (SS) measured a year later was -0.61 [22];CVAFD is generally viewed as an indirect indicator of SS, which is one of the most important wool quality traits but expensive to measure. The results suggest that infusion of ASM genes is an effective approach to improve wool fiber diameter and its coefficient of variation.

#### Wool production traits

CFW is the most important wool production trait to be improved in any Merino breeding strategy. In this study, both heterosis and the true breed differences contributed to changes in CFW and yield. However, the magnitude of superiority presented by ASMs for CFW (11.68%) and yield (14.47%) over GAF sheep were much higher than that of the heterosis expressed (1.72% and -1.42%, respectively, for CFW and yield). The superiority of ASMs for CFW is obviously attributed to its advantage expressed in yield compared with the GAF sheep, which was observed to have higher GFW (1.77%) than the ASMs.

#### Wool staple length

The ASMs sampled in the study were estimated to be shorter in wool staple length (-0.44cm, -4.36%) than purebred GAF sheep. Both breed genetic difference and heterosis (1.39%) contributed to the variation of YSL. The superiority of ASMs for AFD may be the cause to its inferiority in YSL, as AFD is known to have a positive genetic correlation with staple length (0.19[15]). Nevertheless, keeping a 1/4 level of ASM inheritance in a breeding strategy will allow optimal growth of wool staple length, as showed in the AGG genotype. On the other hand, the YSL/AFD ratio is widely used to express the relative growth of wool staple length in a breeding program focused on improving AFD. The estimated 16.17% superiority of ASMs for YSL/AFD ratio over GAF sheep implies that the former were more capable of keeping a relative higher growth in wool staple length while decreasing wool fiber diameter than pure bred GAF sheep.

#### Growth traits

The ASMs sampled in this study were estimated to be genetically inferior to purebred GAF sheep for growth traits. The magnitude of their true breed differences were low (-0.72% to -2.93%) for pre-weaning growth traits and relatively high for YWT (-16.93%) and post-weaning growth rate (-50.97%). Apparently, the low YWT estimates for ASMs in this study were the result of low post-weaning growth. The desirable heterosis estimated in the study can be a remedy to the undesirable breed effect when introducing ASM genes into the GAF sheep gene pool. Inferiority of ASMs for post-weaning growth estimated in this study may generally be due to the rule that the wool fiber diameter is positively genetically correlated with body weight-associated growth traits. The weighted means of estimates in the literature for genetic correlation between fiber diameter and post-weaning body weight and adult body weight are 0.2 and 0.15, respectively [15]. In our study, it may due to the low body weight of the original ASM rams imported. The significant influence of the within-genotype sire effect on the growth traits support the above argument. In the high-and-cold GAF sheep benefiting area, animals with higher body weight and growth are more welcomed than animals with smaller body weight. Accordingly, more emphasis should be placed on body weight selection when importing ASM rams for crossbreeding programs with GAF sheep.

#### Clean fleece weight to metabolic body weight ratio

CFW/YWT^0.75^ has previously been used as an indicator of wool production efficiency [17]. In our study, ASMs were estimated to be 25.34% more efficient in wool production than GAF sheep. The implication of the result is that the ASMs have the genetic makeup to produce finer, hence more valuable, wool with the same feed consumption for maintenance and production as GAF sheep. This characteristic is mostly required for the establishment of an economically efficient and eco-friendly Merino breeding and production system in China.

Precautions have to be taken while using the results derived from our study, since the ASM rams introduced might not be truly representative of Australian Superfine Merinos, and the imported rams were crossed with the GAF nucleus population raised in the environmental and management conditions in the Gansu Provincial Sheep Breeding Technology Extension Station. Therefore, further studies and evaluations are warranted to address this issue.

## Conclusions

The results of this study provide useful information on the potential as well as the method of benefiting from the introduction of ASM genes into the domestic finewool breed gene pool in China. Crossbreeds of 1/2 ASM expressed the most desirable effect for improving AFD, CFW, yield, CVAFD, YSL/AFD, and CFW/YWT^0.75^ but showed the least post-weaning growth rate and YWT. A backcross genotype with 1/4 ASM obtained a moderate improvement for AFD, CFW, CVAFD, YSL/AFD, and CFW/YWT^0.75^ but the highest YSL, prwADG among the three genotypes, and similar YWT and powADG with purebred GAF sheep. There appeared to be a clear improving trend for AFD, CVAFD, YSL/AFD, and CFW/YWT^0.75^ with the increase of ASM inheritance. Except for yield (-1.42%) and CFW/YWT^0.75^ (-1%), the heterosis estimates were generally low and positive and ranged from 0.1% for CVAFD to 4% for powADG. There is potential to improve the relevant traits through exploiting heterosis to a varying extent.

Utilizing heterosis provides alternative remedies to traits like YSL, PowADG, and YWT to which the infusion of ASM genes resulted in undesirable changes. Heterosis estimates provide detailed information for an appropriate crossbreeding program design for the fine wool sheep selection context and allow us to balance the potential benefits from exploiting heterosis and genetic variation between the breeds.

ASMs sampled in this study were demonstrated to be superior to GAF in terms of AFD, CVAFD, yield, and CFW but inferior for YSL, PowADG, and YWT. We also demonstrated that they were more efficient than GAF in wool production and staple length growth. Our study strongly suggests that an infusion of ASM genes is an effective and appropriate approach to improve wool microns as well as wool production in GAF sheep. However, as large body size is more sought after in the finewool sheep industry in the semi-arid, high-and-cold environment in Gansu province, more emphasis should be placed on body weight selection when importing ASM rams and carrying out crossbreeding. In addition, we recommend that the first crossbreeding be carried out in the nucleus population of GAF sheep, and, therefore, replacement breeding rams with 1/2 ASM genotypes be intensively selected and provided for the multiplier flocks and commercial flocks in the whole industry.

Our study identified that the introduction of ASM rams is potentially an effective and viable approach to remedy the paucity of superfine Merino genetic resources while developing superfine Merino breeds in China. Because of the small number, hence the inadequate representation of rams sampled in this study, further research is warranted to verify the conclusions of this study.
